# Annual Death Rate in Atlanta

**Published:** 1884-10

**Authors:** 


### ANNUAL DEATH RATE IN ATLANTA.

The comparative healthfulness of towns and cities is a question of the deepest importance to the inhabitants of them, and of general interest to the medical profession. The annual death rate, which in the absence of epidemics, presents astonishing uniformity, furnishes a reasonably safe standard of comparison. For Atlanta, at an elevation of 1068 feet above the sea level, almost free from malarious influences, remote from swamps or large streams of water, and with remarkable facilities for drainage, a very high degree of exemption from disease has been anticipated. How far those anticipations have been realized, will appear from the following statistics furnished by the efficient Secretary of the Board of Health:

"In 1880 the total mortality was 679; whites 288, colored 391, total mortality over five years, 392, whites 175, colored 217, total mortality under five years 287, whites 113, colored 174, total annual death rate 17.08. In 1881 the total mortality was 962; whites 423, colored 539; total mortality over five years 518; whites, 249, colored, 269. Total mortality under five years 444, whites 174, colored 270. Total annual death rate 24.66. In 1882 the total mortality was 924; whites 360, colored 564; total mortality over five years 497; whites 197, colored 300; total mortality under five years 427, whites 163, colored 264. Total death-rate 21.00. In 1883 the total mortality was 1,087; whites 443, colored 644. Total mortality over five years 554; whites 249, colored 305. Total mortality under five years 533; whites 194, colored 339. Total annual death rate 21.74.

For the first eight months of the current year the following figures are submitted:

The total number of deaths for eight months, ending September 1st, was seven hundred and fifty, of which two hundred and ninety-six were whites and four hundred and fifty-four colored. Three hundred and sixty-five were among children under five years of age; one hundred and thirty-seven white, and two hundred and twenty-eight colored.

If this mortality is taken as an average for the whole year it would give a total annual death rate of 22.50 to the thousand. The rate among the whites 14.80 to the thousand and among the blacks of 34.05 to the thousand. But as four of the healthiest months of the year are the remaining months, the annual death rate will be lessened. Four of the past months May, June, July and August, are the sickliest of the year, the months in which in all cities, and in fact all places, the largest number of deaths occur.

Want of space prevents any extended comment in this issue on the above facts. The vast disproportion in the death rate between the black and white races, is the most obvious and startling truth presented. The dwellings of the former are not confined to any unhealthy quarter, but are scattered all over the city, like those of the latter. All are alike exposed to the same general conditions. The causes of the fearful mortality of the blacks is to be sought in their peculiar personal and domestic habits and conditions, and the hygienic regulations which will be potent to mitigate or destroy it must look beyond the streets and the sewers and enter the domiciles of the exposed and suffering.

